# Perinatal Outcomes Among Patients With Sepsis During Pregnancy

**DOI:** 10.1001/jamanetworkopen.2021.24109

**Published:** 2021-09-03

**Authors:** Christine A. Blauvelt, Kiana C. Nguyen, Arianna G. Cassidy, Stephanie L. Gaw

**Affiliations:** 1 Department of Obstetrics, Gynecology and Reproductive Sciences, Division of Maternal-Fetal Medicine, University of California, San Francisco

## Abstract

**Importance:**

Rates of maternal sepsis are increasing, and prior studies of maternal sepsis have focused on immediate maternal morbidity and mortality associated with sepsis during delivery admission. There are no data on pregnancy outcomes among individuals who recover from their infections prior to delivery.

**Objective:**

To describe perinatal outcomes among patients with antepartum sepsis who did not deliver during their infection hospitalization.

**Design, Setting, and Participants:**

This retrospective cohort study was conducted using data from August 1, 2012, to August 1, 2018, at an academic referral center in San Francisco, California. Included patients were all individuals with nonanomalous, singleton pregnancies who delivered after 20 weeks’ gestation during the study period. Data were analyzed from March 2020 through March 2021.

**Exposures:**

Antepartum admission for infection with clinical concern for sepsis and hospital discharge prior to delivery.

**Main Outcomes and Measures:**

The primary outcome was a composite of perinatal outcomes associated with placental dysfunction and consisted of 1 or more of the following: fetal growth restriction, oligohydramnios, hypertensive disease of pregnancy, cesarean delivery for fetal indication, child who is small for gestational age, or stillbirth.

**Results:**

Among 14 565 patients with nonanomalous singleton pregnancies (mean [SD] age at delivery, 33.1 [5.2] years), 59 individuals (0.4%) were in the sepsis group and 14 506 individuals (99.6%) were in the nonsepsis group; 8533 individuals (59.0%) were nulliparous. Patients with sepsis, compared with patients in the reference group, were younger (mean [SD] age at delivery, 30.6 [5.7] years vs 33.1 [5.2] years; *P* < .001), were more likely to have pregestational diabetes (5 individuals [8.5%] vs 233 individuals [1.6%]; *P* = .003), and had higher mean (SD) pregestational body mass index scores (26.1 [6.1] vs 24.4 [5.9]; *P* = .03). In the sepsis group, the most common infections were urinary tract infections (24 patients [40.7%]) and pulmonary infections (22 patients [37.3%]). Among patients with sepsis, 5 individuals (8.5%) were admitted to the intensive care unit, the mean (SD) gestational age at infection was 24.6 (9.0) weeks, and the median (interquartile range) time from infection to delivery was 82 (42-147) days. Antepartum sepsis was associated with higher odds of placental dysfunction (21 patients [35.6%] vs 3450 patients [23.8%]; odds ratio, 1.77; 95% CI, 1.04-3.02; *P* = .04). On multivariable logistic regression analysis, antepartum sepsis was an independent factor associated with placental dysfunction (adjusted odds ratio, 1.88; 95% CI, 1.10-3.23; *P* = .02) after adjusting for possible confounders.

**Conclusions and Relevance:**

This study found that pregnancies complicated by antepartum sepsis were associated with higher odds of placental dysfunction. These findings suggest that increased antenatal surveillance should be considered for these patients.

## Key Points

**Question:**

Do patients who remain pregnant after discharge from an antepartum sepsis hospitalization have increased rates of perinatal complications?

**Findings:**

In this cohort study of 14 565 patients with nonanomalous, singleton pregnancies, a history of antepartum sepsis hospitalization was associated with higher odds of perinatal complications that are associated with placental dysfunction compared with no history of antepartum sepsis. After adjustment for possible confounders, including maternal age, parity, body mass index, and medical comorbidities, patients with antepartum sepsis had 2-fold higher odds of perinatal complications.

**Meaning:**

This study found that pregnancies with antepartum sepsis were associated with higher odds of complications that are associated with placental dysfunction, suggesting that increased antenatal surveillance may be warranted for these patients.

## Teaser

This cohort study compares rates of perinatal complications among pregnant individuals with and without antepartum sepsis hospitalization.

## Introduction

Maternal sepsis is the second leading cause of maternal mortality in the United States.^1^ Among all maternal deaths in the US, 13% are attributed to infection or sepsis, with case fatality rates as high as 10% to 30% in the obstetric population.^2,3,4^ Pregnant patients may be particularly susceptible to rapid progression to sepsis and septic shock due to pregnancy-specific physiologic, mechanical, and immunological adaptations.^5^ Rates of sepsis during pregnancy and the puerperium are also increasing, with 1 population-based study in Texas^6^ finding a 2-fold increase in pregnancy-associated sepsis, from 11 incidences per 10 000 deliveries in 2001 to 26 incidences per 10 000 deliveries in 2010. This increase may be associated with evolving characteristics of obstetric patients, including older age, increased body mass index scores (BMI; calculated as weight in kilograms divided by height in meters squared), and increasing prevalence of chronic comorbid conditions.

Pregnancies complicated by sepsis are associated with increased rates of adverse obstetric outcomes, including cesarean delivery, postpartum hemorrhage, and preterm delivery.^7,8,9^ Much of our current understanding of sepsis in pregnancy is based on studies of peripartum infections occurring during labor or the postpartum period, such as septic abortion, intra-amniotic infection, and endometritis.^10,11,12,13^ Studies of antepartum infections have primarily focused on immediate maternal outcomes, such as intensive care unit (ICU) admission and death. Little is known about the association of sepsis with long-term pregnancy outcomes, particularly among individuals who recover from their infections prior to delivery.

There is abundant evidence regarding placental dysfunction after maternal infection with pathogens capable of causing placental or fetal infection.^14,15,16,17,18,19^ Some studies have also suggested an association between infection with pathogens that do not cross the utero-placental interface and placental dysfunction.^20,21^ Antenatal risk stratification for placental disorders is of paramount importance, given that impaired placental development and function are important factors associated with multiple pregnancy complications, including preeclampsia, placental abruption, preterm birth, infants born small for their gestational age, and stillbirth.

The objective of this study was to describe perinatal outcomes among patients with an antepartum sepsis hospitalization who recovered from their infection and did not deliver prior to hospital discharge. We hypothesized that these patients were at an increased risk of pregnancy complications, particularly complications associated with placental dysfunction.

## Methods

The University of California, San Francisco, institutional review board approved this retrospective cohort study with a waiver of informed consent because of the study’s retrospective design and use of deidentified data. The Strengthening the Reporting of Observational Studies in Epidemiology (STROBE) reporting guideline was followed in the writing of this report.

### Study Population

We performed a retrospective cohort study of patients with a nonanomalous, singleton pregnancy who delivered at 20 weeks’ gestation or later at an academic referral center from August 1, 2012, to August 1, 2018. Individuals included in the study were identified through the institution’s electronic medical and delivery records. Patients were screened using diagnoses coded with the *International Classification of Diseases, Ninth Revision, Clinical Modification *(*ICD-9-CM*) and *International Statistical Classification of Diseases, Tenth Revision, Clinical Modification *(*ICD-10-CM*) for an infection-related hospital admission during the 10 months prior to delivery (see eMethods in the Supplement for list of *ICD-9-CM* and *ICD-10-CM* codes used). Exclusion criteria included toxoplasmosis, syphilis, varicella, parvovirus B19, Zika virus, rubella, cytomegalovirus, and herpes simplex virus (TORCH) infections. All patients identified through *ICD-9-CM* and *ICD-10-CM* codes were reviewed by 2 authors (C.A.B. and K.C.N.) to confirm the presence of an antepartum infection and to determine whether there was clinical concern for sepsis. We defined sepsis as “life-threatening organ dysfunction caused by a dysregulated host response to infection,”^22^ in accordance with The Third International Consensus Definitions for Sepsis and Septic Shock Task Force. We defined the antepartum period as the time from a positive pregnancy test until onset of labor or rupture of membranes. Patients were included in the sepsis group if they had an antepartum infection with clinical concern for sepsis and were subsequently discharged prior to delivery. Patients were included in the reference group if they did not have an infection with concern for sepsis during their pregnancy.

### Variables and Outcome Measures

We extracted patient variables from medical records. Collected variables were maternal age, parity, pregestational BMI (patient-reported BMI immediately prior to pregnancy or earliest documented BMI during pregnancy), pregestational diabetes (ie, type 1 or 2 diabetes diagnosed prior to pregnancy or hemoglobin A_1C_ ≥6.5% prior to 14 weeks’ gestation [to convert to proportion of total hemoglobin, multiply by 0.01]), gestational diabetes (ie, abnormal glucose tolerance testing after 14 weeks’ gestation by 1-step or 2-step approach), chronic hypertension (ie, elevated blood pressure diagnosed prior to pregnancy or before 20 weeks’ gestation), smoking during pregnancy, and admission indication during delivery hospitalization.

We additionally obtained perinatal outcomes. Collected outcomes were fetal growth restriction (ie, sonographic estimation of fetal weight <10th percentile for gestational age), oligohydramnios (ie, amniotic fluid index ≤5 cm or single deepest pocket <2 cm), hypertensive disease of pregnancy (ie, new hypertension after 20 weeks’ gestation, with systolic blood pressure ≥140 mm Hg or diastolic blood pressure ≥90 mm Hg on 2 occasions at least 4 hours apart), cesarean delivery for nonreassuring fetal status or intolerance of labor, infant born small for gestational age (ie, sex-specific birthweight <10th percentile for gestational age),^23^ stillbirth, preterm birth, preterm premature rupture of membranes at less than 37 weeks’ gestation, intra-amniotic infection (ie, maternal fever and ≥1 of fetal tachycardia, maternal white blood cell count >15,000/mm^3^, or purulent fluid from the cervical os), retained placenta, cesarean delivery for any indication, postpartum hemorrhage (ie, delivery blood loss ≥1000 mL), blood transfusion during delivery hospitalization, postpartum infection (ie, uterine or surgical site infection diagnosed up until 6 weeks post partum), maternal ICU admission during delivery hospitalization, 5-minute Apgar score less than 7, and term neonatal ICU admission.

Patient characteristics and perinatal outcomes were compared between the sepsis group and reference group. The primary outcome was a composite of perinatal outcomes associated with placental dysfunction and consisted of 1 or more of the following: fetal growth restriction, oligohydramnios, hypertensive disease of pregnancy, cesarean delivery for nonreassuring fetal status or intolerance of labor, infant born small for gestational age, or stillbirth. Secondary outcomes included each component of the composite placental dysfunction outcome, as well as other maternal and neonatal complications.

### Statistical Analysis

Descriptive analyses were performed for all patient characteristics and clinical variables. Between-group differences were assessed by χ^2^ test or Fisher exact test for binary variables and by *t* test for continuous variables. Subgroup analyses were performed according to severity of infection, gestational age at time of infection, and organ system involved. Multivariable logistic regression analysis was performed to adjust for potential confounders that could be associated with placental dysfunction. All statistical tests were 2-tailed, with *P* values less than .05 considered statistically significant. Statistical analyses were performed using Python programming language version 3.8.5 (Python Software Foundation) and the following libraries: SciPy version 1.5.2 (Travis Oliphant, Pearu Peterson, Eric Jones) and Pandas version 1.1.2 (NumFocus). Data were analyzed from March 2020 through March 2021.

## Results

**Figure 1.**
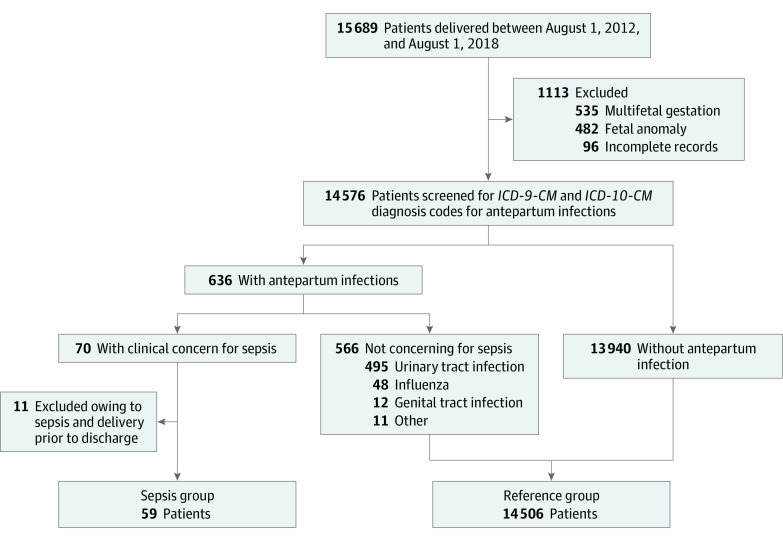
Patient Flowchart *ICD-9-CM* indicates *International Classification of Diseases, Ninth Revision, Clinical Modification*; *ICD-10-CM*, *International Statistical Classification of Diseases, Tenth Revision, Clinical Modification*.

Among 14 565 patients (mean [SD] age 33.1 [5.2] years), 59 individuals (0.4%) were in the sepsis group and 14 506 individuals (99.6%) were in the nonsepsis group; 8533 individuals (59.0%) were nulliparous. Figure 1 shows the flow of patients. There were 15 689 patients who delivered at our institution from August 1, 2012, to August 1, 2018. Of these, 1113 patients were excluded: 535 patients excluded for multifetal gestation, 482 excluded for major fetal structural or genetic anomalies, and 96 excluded for missing data. We cross-referenced the remaining 14 576 patients with hospital discharge records to identify patients with an infection in the 10 months prior to delivery, resulting in 700 patients. The medical records for these patients were then reviewed in detail, with 636 patients (4.4%) confirmed to have an antepartum infection and 70 patients (0.5%) with sepsis; 10 of these patients (14.3%) required ICU admission. We excluded 11 patients (15.7%) who delivered during their sepsis hospitalization. Among individuals with antepartum sepsis hospitalizations, 59 patients (84.3%) were discharged prior to delivery and were included in the sepsis group.

Table 1 summarizes the baseline characteristics of patients in the sepsis and reference groups. Patients in the sepsis group were younger (mean [SD] age at delivery, 30.6 [5.7] years vs 33.1 [5.2] years; *P* < .001), were more likely to have pregestational diabetes (5 individuals [8.5%] vs 233 individuals [1.6%]; *P* = .003), and had higher mean (SD) pregestational BMI scores (26.1 [6.1] vs 24.4 [5.9]; *P* = .03) than patients in the reference group. Among patients in the sepsis group, the most common infection types were urinary tract infections (24 patients [40.7%]) and pulmonary infections (22 patients [37.3%]), and 5 individuals (8.5%) were admitted to the ICU. The mean (SD) gestational age at the time of infection was 24.6 (9.0) weeks, and the median (interquartile range) time from infection diagnosis to delivery was 82 (42-147) days. Quick Sequential (Sepsis-Related) Organ Failure Assessment (qSOFA) scores of 2 points or more were found for 16 patients (27.1%), and 5 patients (8.5%) were admitted to the ICU. In the sepsis group, 1 patient died post partum, giving an overall maternal mortality rate within this group of 1.7%. This patient had a history of cystic fibrosis and developed sepsis from pneumonia, recovered, and later died from noninfectious pulmonary complications.

**Table 1.** Characteristics of Patient Groups

Characteristic	Patients, No. (%)		*P* value
	Sepsis group (n = 59)	Reference group (n = 14 506)	
Age at delivery, mean (SD), y	30.6 (5.7)	33.1 (5.2)	<.001
Nulliparous	28 (47.5)	8505 (58.6)	.09
Pregestational BMI, mean (SD)	26.1 (6.1)	24.4 (5.9)	.03
Pregestational diabetes	5 (8.5)	233 (1.6)	.003
Gestational diabetes	12 (20.3)	2450 (16.9)	.49
Chronic hypertension	2 (3.4)	615 (4.2)	>.99
Smoking during pregnancy	2 (3.4)	451 (3.1)	.71
Delivery admission indication			
Labor or term ruptured membranes	40 (67.8)	10 169 (70.1)	.83
Induction of labor	13 (22.0)	2899 (20.0)	
Scheduled cesarean delivery	6 (10.3)	1438 (9.8)	
Gestational age, mean (SD), wk			
At delivery	38.6 (2.1)	39.1 (2.4)	.13
At infection	24.6 (9.0)	NA	NA
Latency from infection to delivery, median (IQR), d	82 (43-147)	NA	NA
Infection types			
Urinary tract or kidney	24 (40.7)	NA	NA
Pulmonary	22 (37.3)		
Gastrointestinal	3 (5.1)		
Periodontal	1 (1.7)		
Sepsis not otherwise specified	9 (15.3)		
Antepartum ICU admission	5 (8.5)	NA	NA
Antepartum hospital length of stay, median (IQR), d	2 (2-3.5)	NA	NA

Abbreviations: BMI, body mass index (calculated as weight in kilograms divided by height in meters squared); ICU, intensive care unit; IQR, interquartile range; NA, not applicable.

**Figure 2.**
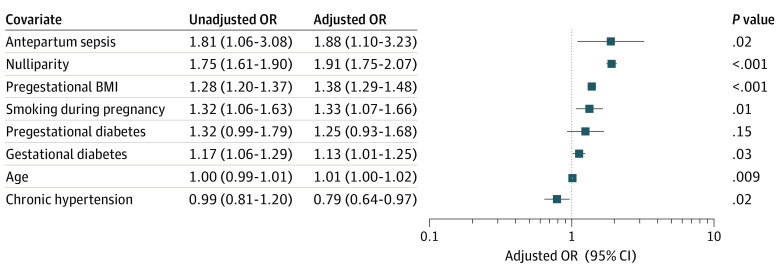
Multivariable and Univariable Logistic Regression for Placental Dysfunction Data for the univariable analysis indicate odds of having placental dysfunction for each covariate using simple logistic regression; data for the multivariable analysis indicate adjusted odds ratios (ORs). All variables are covariates retained in the final model after adjustment. BMI indicates body mass index.

Perinatal outcomes are presented in Table 2. Features of placental dysfunction were more commonly seen among patients in the sepsis group than patients in the reference group (21 patients [35.6%] vs 3450 patients [23.8%]; odds ratio [OR], 1.77; 95% CI, 1.04-3.02; *P* = .04). Antepartum sepsis was associated with higher odds of hypertensive disease of pregnancy (14 patients [23.7%] vs 1614 patients [11.1%]; OR, 2.49; 95% CI, 1.36-4.54; *P* = .006). Among patients with sepsis, 5 individuals (8.5%) developed preeclampsia and 9 individuals (15.3%) developed gestational hypertension (64.3% of those with sepsis who had hypertensive disease of pregnancy). Antepartum sepsis was also associated with higher odds of postpartum hemorrhage (14 patients [23.7%] vs 1871 patients [12.9%]; OR, 2.10; 95% CI, 1.15-3.83; *P* = .02) and maternal ICU admission during delivery hospitalization (2 patients [3.4%] vs 10 patients [0.1%]; OR, 39.70; 95% CI, 8.51-185.24; *P* = .002). Other perinatal outcomes were similar between the 2 groups. In the multivariable logistic regression analysis (Figure 2), antepartum sepsis was an independent factor associated with placental dysfunction (adjusted OR [aOR], 1.88; 95% CI, 1.10-3.23; *P* = .02) after adjustment for possible confounders. Parity, pregestational BMI, smoking during pregnancy, gestational diabetes, age, and chronic hypertension were also factors associated with placental dysfunction in multivariable analysis.

**Table 2.** Perinatal Outcomes

Outcome	Patients, No. (%)		OR (95% CI)^a^	*P* value
	Sepsis group (n = 59)	Reference group (n = 14 506)		
Outcome associated with placental dysfunction				
Fetal growth restriction	1 (5.6)	154 (3.3)	1.72 (0.23-12.97)	.46
Oligohydramnios	0	141 (3.0)	NA	NA
Hypertensive disease of pregnancy	14 (23.7)	1614 (11.1)	2.49 (1.36-4.54)	.006
Cesarean delivery for nonreassuring fetal status or intolerance of labor	4 (6.9)	639 (4.4)	1.60 (0.58-4.44)	.32
Small for gestational age	7 (11.9)	1438 (9.9)	1.22 (0.55-2.70)	.66
Stillbirth	0	91 (0.6)	NA	NA
Composite placental dysfunction (≥1 of the previous outcomes)	21 (35.6)	3450 (23.8)	1.77 (1.04-3.02)	.04
Secondary perinatal outcomes				
Preterm birth				
<37 wk	6 (10.2)	1495 (10.3)	0.99 (0.42-2.30)	>.99
<34 wk	3 (5.1)	572 (3.9)	1.31 (0.41-4.18)	.51
Preterm premature rupture of membranes	3 (5.1)	413 (2.8)	1.83 (0.57-5.86)	.24
Intra-amniotic infection	2 (3.4)	1438 (5.7)	0.58 (0.14-2.39)	.77
Retained placenta	6 (10.3)	1390 (10.0)	1.04 (0.44-2.42)	.83
Cesarean delivery	20 (33.9)	3484 (24.0)	1.62 (0.95-2.79)	.09
Postpartum hemorrhage	14 (23.7)	1871 (12.9)	2.10 (1.15-3.83)	.02
Blood transfusion	5 (8.5)	563 (3.9)	2.29 (0.91-5.75)	.08
Postpartum infection	1 (1.7)	826 (5.7)	0.29 (0.04-2.06)	.26
Maternal ICU admission at delivery	2 (3.4)	10 (0.1)	39.7 (8.5-185.2)	.002
5-min Apgar score <7	1 (1.7)	785 (5.4)	0.30 (0.04-2.18)	.38
Term neonatal ICU admission	3 (5.1)	1417 (9.8)	0.49 (0.15-1.58)	.28

Abbreviation: ICU, intensive care unit; OR, odds ratio.

^a^ ORs were calculated using the χ^2^ or Fisher exact test.

In subgroup analyses (Table 3), among patients with sepsis vs those without sepsis, earlier gestational age at the time of infection was associated with higher odds of placental dysfunction (12 of 26 patients [46.2%] at <24 weeks; OR, 2.75; 95% CI, 1.27-5.94; *P* = .02), but later gestational age at time of infection was not (9 of 33 patients [27.3%] at ≥24 weeks’ gestation; OR, 1.20; 95% CI, 0.56-2.59; *P* = .68). Patients with antepartum sepsis prior to 24 weeks were more likely to develop hypertensive disease of pregnancy (8 patients [30.8%] vs 1614 patients [11.1%]; OR, 3.55; 95% CI, 1.54-8.18; *P* = .01) and to have newborns who were small for their gestational age (6 patients [23.1%] vs 1438 patients [9.9%]; OR, 2.73; 95% CI, 1.09-6.80; *P* = .04) compared with patients in the reference group who did not have antepartum sepsis. Patients with antepartum sepsis prior to 24 weeks also delivered newborns with lower mean (SD) birth weights than patients in the reference group (2967 [537] g vs 3249 [620] g; *P* = .02). There were no differences in outcomes when stratified by organ system involved, antepartum ICU admission, or qSOFA score of 2 or greater.

**Table 3.** Placental Dysfunction by Organ System, Severity, and Timing of Infection

Risk factor	Total patients, No.	Patients with placental dysfunction, No. (%)	OR (95% CI)	*P* value
Reference group	14 506	3450 (23.8)	1 [Reference]	NA
Sepsis group	59	21 (35.6)	1.77 (1.04-3.02)	.04
Organ system involved				
Kidney	24	8 (33.3)	1.60 (0.69-3.75)	.34
Pulmonary	22	9 (40.9)	2.22 (0.95-5.19)	.08
Gastrointestinal	3	0	NA	NA
Other	10	4 (40.0)	2.14 (0.60-7.58)	.26
Severity of infection				
Antepartum ICU admission	5	1 (20.0)	0.80 (0.09-7.15)	>.99
qSOFA score ≥2	16	5 (31.2)	1.46 (0.51-4.20)	.56
Gestational age at infection, wk				
0-24	26	12 (46.2)	2.75 (1.27-5.94)	.02
≥24	33	9 (27.3)	1.20 (0.56-2.59)	.68

Abbreviations: ICU, intensive care unit; NA, not applicable; OR, odds ratio; qSOFA, quick Sequential (Sepsis-Related) Organ Failure Assessment.

Placental pathology results were available for 20 patients (33.9%) and unavailable for 39 patients (66.1%) in the sepsis group. We were therefore unable to find associations between histopathological findings and clinical outcomes of placental dysfunction. Among individuals in the sepsis group, 41 patients (69.5%) had a third-trimester ultrasonography for fetal growth. Among 7 patients (11.9%) with sepsis who delivered infants who were small for their gestational age, 4 patients (57.1%) had third-trimester ultrasonography screening for fetal growth restriction and 1 patient (14.3%) was diagnosed with fetal growth restriction prenatally.

## Discussion

This cohort study found that patients with a history of antepartum sepsis had statistically significantly higher odds of obstetric complications associated with placental dysfunction. When adjusting for possible confounders, including maternal age, parity, BMI score, and medical comorbidities, patients with antepartum sepsis had nearly 2-fold higher odds of placental dysfunction compared with patients without antepartum sepsis. The timing of infection during pregnancy also had important associations with perinatal outcomes. Early infection (ie, at less than 24 weeks’ gestational age) was associated with the greatest increase in odds of composite placental dysfunction, hypertensive disease of pregnancy, and newborns who were small for gestational age. High rates of obstetric complications were also seen in those who experienced antepartum sepsis, including postpartum hemorrhage, blood transfusion, cesarean delivery, and peripartum ICU admission.

Consistent with previous work,^4,8,24^ our study found that 70 pregnant patients (0.5%) had antepartum sepsis, with 10 of these patients (14.3%) requiring maternal ICU admission and 11 patients (15.7%) having imminent delivery. Much of the previous literature regarding severe antepartum infection has focused on maternal morbidity, and few studies have examined obstetric and neonatal outcomes. A population-based cohort study^25^ in Nova Scotia, Canada, compared pregnant patients with and without hospital admission for respiratory illness and reported increased rates of infants who were born small for their gestational age and decreased birth weights among patients with respiratory hospitalization. Our study had similar findings among patients with antepartum sepsis, although our data were statistically significant only among individuals with sepsis whose infections occurred at less than 24 weeks’ gestation vs those without sepsis (infants born small for their gestational age: 6 patients [23.1%] vs 1438 patients [9.9%]; mean [SD] birthweight 2967 [537] g vs 3249 [620] g). In another study, Farkash et al^26^ found that antepartum pyelonephritis was associated with multiple perinatal outcomes that are potentially associated with placental dysfunction, including fetal growth restriction, placental abruption, preterm delivery, intrapartum fetal distress, and an Apgar score less than 7 at 1 minute. In contrast to our findings, their study did not find a difference in rates of hypertensive disorders among individuals with and without antepartum pyelonephritis.

Our study differs from previous investigations in several important ways. First, this is the first study, to our knowledge, to examine perinatal outcomes among patients who recovered from antepartum sepsis prior to delivery, given that prior studies of maternal sepsis have focused on sepsis that occurred during delivery admission. This represents a distinct clinical scenario from antepartum sepsis that leads to imminent delivery. Second, we did not restrict our study by organ system involved in the infection and instead included all infection types that led to sepsis. Third, we conducted detailed medical record reviews for patients with antepartum infections, and only individuals with concerning features for sepsis were included. Fourth, our study was unique, to our knowledge, in examining associations of gestational age at the time of infection and of latency between infection and delivery with perinatal outcomes.

We hypothesize that the dysregulated host response to maternal infection that occurs during sepsis may disrupt placental development and function, leading to poor perinatal outcomes among these patients. There is a large body of research finding that placental pathologies, such as maternal spiral artery remodeling, abnormal villous development, maternal vascular malperfusion, and impaired umbilical blood flow, are associated with pregnancy complications.^27,28,29,30,31^ Moreover, abnormal placental histopathologic findings have been observed among pregnant individuals with bacterial, viral, and parasitic infections.^32,33,34,35,36^ Maternal infection and systemic inflammation may be associated with impaired vasculogenesis and angiogenesis, with associated placental insufficiency, inadequate oxygen and nutrient transport to the fetus, and adverse birth outcomes. These outcomes may be magnified among pregnant individuals with sepsis at earlier gestational ages. We hypothesize that this association may be explained by the coincident timing of infection with establishment of placental vasculature or by increased duration of exposure to utero-placental dysfunction.

The recognition that antepartum sepsis may be associated with placental dysfunction has important implications for pregnancy management. Ultrasonographic screening for fetal growth restriction may be considered for patients with a history of antepartum sepsis. Of 7 patients in our study who delivered infants who were small for their gestational age, 4 patients (57.1%) had growth ultrasonography screenings in the third trimester and 1 patient (14.3%) was diagnosed with fetal growth restriction prenatally. Fetal growth restriction is known to be associated with increased risk of stillbirth, neonatal morbidity, and mortality,^37^ underscoring the potential need for increased antepartum surveillance for pregnancies complicated by antepartum sepsis. We also observed increased rates of hypertensive disease of pregnancy in the sepsis group, occurring in approximately 24% of patients. Of note, most of these individuals (9 patients [64.3%]) exhibited mild hypertensive disease at the time of delivery. These findings suggest that patients with antepartum sepsis should be counseled on signs and symptoms of preeclampsia and receive close monitoring of their blood pressure.

### Strengths and Limitations

Strengths of our study included that, to our knowledge, this is the first study to directly assess perinatal outcomes of patients who experienced sepsis in pregnancy and recovered prior to delivery. Additionally, we performed detailed medical record review, allowing us to have complete and accurate data for sepsis admission and ultimate delivery admission.

Several limitations of our work should be recognized, including the retrospective nature of data collection at a single institution. We included only pregnancies that led to delivery at our institution; thus, patients who delivered elsewhere may have been missed. If such patients had a different risk of perinatal outcomes than those who delivered at our institution, this could have biased our findings. Furthermore, our sample size was likely too small to detect differences in rare perinatal complications, such as stillbirth. In addition, we did not use standardized diagnostic criteria for sepsis, given that pregnant patients have been excluded from major trials validating systemic inflammatory response syndrome (SIRS), SOFA, qSOFA, and other sepsis scoring systems. Therefore, these sepsis severity scores have not been well validated in obstetric populations and have not been adjusted for pregnancy physiology. Given the lack of a consensus definition of sepsis in the obstetric population, patients in this study were included in the sepsis group if there was agreement between 2 authors (C.A.B. and K.C.N.) regarding the presence of infection and organ dysfunction. Additionally, placental pathology reports were not available for most patients (39 patients [66.1%]) in the sepsis group. It is likely that many placentas were not sent for histopathologic review because the patients had recovered from sepsis at the time of delivery. Thus, we were not able to associate clinical findings of placental dysfunction with histopathologic findings; however, this should be considered in future work.

## Conclusions

This study found that antepartum sepsis was associated with increased odds of placental dysfunction. Earlier gestational age at the time of infection was associated with higher odds of placental dysfunction, including hypertensive disease of pregnancy and lower birth weight. Our study provides new information to inform patient counseling regarding expected perinatal outcomes after antepartum sepsis and the potential need for increased pregnancy surveillance. This study may also open up new avenues for research regarding pathophysiologic mechanisms of placental dysfunction after antepartum sepsis and potential interventions including anti-inflammatory medications to mediate the association of sepsis with adverse pregnancy outcomes.

## Notes

Supplement. **eMethods.** Coded Diagnoses for Infections During Pregnancy
