# Supplementary material for: Perinatal Outcomes Among Patients With Sepsis During Pregnancy
Source: JAMA Netw Open. 2021 Sep 3;4(9):e2124109. doi: 10.1001/jamanetworkopen.2021.24109 (PMC8417755; doi:10.1001/jamanetworkopen.2021.24109)
Supplement: Supplement. — eMethods. Coded Diagnoses for Infections During Pregnancy [file jamanetwopen-e2124109-s001.pdf]

Supplemental Online Content

Blauvelt CA, Nguyen KC, Cassidy AG, Gaw SL. Perinatal outcomes among patients with sepsis during pregnancy. *JAMA Netw Open*. 2021;4(9):e2124109. doi:10.1001/jamanetworkopen.2021.24109

eMethods. Coded Diagnoses for Infections During Pregnancy

This supplemental material has been provided by the authors to give readers additional information about their work.

© 2021 Blauvelt CA et al. *JAMA Network Open.*

eMethods. Coded Diagnoses for Infections During Pregnancy

International Classification of Diseases, Ninth Revision (ICD-9) Code	***Diagnosis***
038	Septicemia
038.0	Streptococcal septicemia
038.1	Staphylococcal septicemia
038.10	Staphylococcal septicemia, unspecified
038.11	Methicillin susceptible Staphylococcus aureus septicemia
038.12	Methicillin resistant Staphylococcus aureus septicemia
038.19	Other staphylococcal septicemia
038.2	Pneumococcal septicemia [Streptococcus pneumoniae septicemia]
038.3	Septicemia due to anaerobes
038.4	Septicemia due to other gram-negative organisms
038.40	Septicemia due to gram-negative organism, unspecified
038.41	Septicemia due to hemophilus influenzae [H. Influenzae]
038.42	Septicemia due to escherichia coli [E. Coli]
038.43	Septicemia due to pseudomonas
038.44	Septicemia due to serratia
038.49	Other septicemia due to gram-negative organisms
038.8	Other specified septicemias
038.9	Unspecified septicemia
112.5	Candida septicemia (disseminated)
114	Coccidioidomycosis
114.0	Primary coccidioidomycosis (pulmonary)
487	Influenza
487.0	Influenza with pneumonia
487.1	Influenza with other respiratory manifestations
487.8	Influenza with other manifestations
590.0	Chronic pyelonephritis
590.1	Acute pyelonephritis
590.8	Pyelonephritis NOS
646.6	Urinary tract infection complicating pregnancy
646.8	Other specified complications of pregnancy
647.6	HIV complicating pregnancy

© 2021 Blauvelt CA et al. *JAMA Network Open.*

648.9	Other current conditions complicating pregnancy childbirth or the puerperium
658.4	Infection of amniotic cavity
670.2	Puerperal sepsis
760.1	Pyelonephritis in pregnancy affecting fetus or newborn
780.61	Fever presenting with conditions classified elsewhere
785.52	Septic shock
995.9	Systemic inflammatory response syndrome (SIRS)
995.91	Sepsis

International Statistical Classification of Diseases, Tenth Revision, Clinical Modification (ICD-10-CM) Code	***Diagnosis***
A32.7	Listerial sepsis
A40.3	Sepsis due to Streptococcus pneumoniae
A40.9	Streptococcal sepsis, unspecified
A41.01	Sepsis due to Methicillin susceptible Staphylococcus aureus
A41.02	Sepsis due to Methicillin resistant Staphylococcus aureus
A41.1	Sepsis due to other specified staphylococcus
A41.2	Sepsis due to unspecified staphylococcus
A41.3	Sepsis due to Hemophilus influenzae
A41.4	Sepsis due to anaerobes
A41.50	Gram-negative sepsis, unspecified
A41.51	Sepsis due to Escherichia coli [E. coli]
A41.52	Sepsis due to Pseudomonas
A41.53	Sepsis due to Serratia
A41.59	Other Gram-negative sepsis
A41.89	Other specified sepsis
A41.9	Sepsis, unspecified organism
B37.7	Candidal sepsis
B38.0	Acute pulmonary coccidioidomycosis
J10.1	Influenza due to other identified influenza virus with other respiratory manifestations
J11.00	Influenza due to unidentified influenza virus with unspecified type of pneumonia
J11.1	Influenza due to unidentified influenza virus with other respiratory manifestations
J11.2	Influenza due to unidentified influenza virus with gastrointestinal manifestations

© 2021 Blauvelt CA et al. *JAMA Network Open.*

J11.81	Influenza due to unidentified influenza virus with encephalopathy
J11.89	Influenza due to unidentified influenza virus with other manifestations
J12.9	Viral pneumonia, unspecified
N10	Acute pyelonephritis
N15.1	Renal and perinephric abscess
O23.0	Infections of kidney in pregnancy
O23.3	Infections of other parts of urinary tract in pregnancy
O23.4	Unspecified infection of urinary tract in pregnancy
O23.5	Infections of the genital tract in pregnancy
O23.51	Infection of cervix in pregnancy
O23.511	Infections of cervix in pregnancy, first trimester
O23.512	Infections of cervix in pregnancy, second trimester
O23.513	Infections of cervix in pregnancy, third trimester
O23.519	Infections of cervix in pregnancy, unspecified trimester
O23.52	Salpingo-oophoritis in pregnancy
O23.529	Salpingo-oophoritis in pregnancy, unspecified trimester
O23.59	Infection of other part of genital tract in pregnancy
O23.599	Infection of other part of genital tract in pregnancy, unspecified trimester
O75.3	Other infection during labor
O98	Maternal infectious and parasitic diseases classifiable elsewhere but complicating pregnancy, childbirth and the puerperium
O98.43	Viral hepatitis complicating the puerperium
O98.511	Other viral diseases complicating pregnancy, first trimester
O98.512	Other viral diseases complicating pregnancy, second trimester
O98.513	Other viral diseases complicating pregnancy, third trimester
O98.52	Other viral diseases complicating childbirth
O98.53	Other viral diseases complicating the puerperium
O98.7	Human immunodeficiency virus [HIV] disease complicating pregnancy, childbirth and the puerperium
O98.719	Human immunodeficiency virus [HIV] disease complicating pregnancy, unspecified trimester
O98.72	Human immunodeficiency virus [HIV] disease complicating childbirth
O98.919	Unspecified maternal infectious and parasitic disease complicating pregnancy, unspecified trimester
P00.1	Newborn affected by maternal renal and urinary tract diseases
R50.81	Fever presenting with conditions classified elsewhere
R50.9	Fever, unspecified
R65.2	Severe sepsis
R65.20	Severe sepsis without septic shock
R65.21	Severe sepsis with septic shock

© 2021 Blauvelt CA et al. *JAMA Network Open.*

R78.81	Bacteremia

© 2021 Blauvelt CA et al. *JAMA Network Open.*
